# Diagnostic value of bedside lung ultrasound and 12-zone score in the 65 cases of neonatal respiratory distress syndrome and its severity

**DOI:** 10.1186/s12938-024-01224-0

**Published:** 2024-03-06

**Authors:** Peipei Huang, Deng Chen, Xiuxiang Liu, Xiang Zhang, Xiazi Song

**Affiliations:** 1 Department of Ultrasound, The Third Affiliated Hospital of Shanghai University, Wenzhou People’s Hospital, No. 299, Gu’an Road, Ouhai District, Wenzhou, 325000 Zhejiang China; 2 Department of Neonatology, The Third Affiliated Hospital of Shanghai University, Wenzhou People’s Hospital, Wenzhou, Zhejiang China

**Keywords:** Lung ultrasound, Neonatal respiratory distress syndrome, Severity of illness, Mechanical ventilation, Predictive value

## Abstract

**Objective:**

To explore the predictive value of bedside lung ultrasound score in the severity of neonatal respiratory distress syndrome (NRDS) and mechanical ventilation and extubation.

**Methods:**

The clinical data of 65 neonates with NRDS and invasive mechanical ventilation diagnosed in the neonatal intensive care unit of our hospital from July 2021 to July 2022 were retrospectively analyzed. 65 neonates were included in the NRDS group, and 40 neonates with other common lung diseases were selected as the other lung disease groups. All neonates underwent lung ultrasound and X-ray examination. The correlation between lung ultrasound scores and arterial blood gas indexes was analyzed by Pearson. The efficacy of successful evacuation of mechanical ventilation was evaluated by lung ultrasound analysis by ROC curve analysis.

**Results:**

The positive rates of lung consolidation and white lung in NRDS group were higher than the other lung disease groups (*P* < 0.05). The positive rates of bronchial inflation sign and double lung points were lower than these in the other lung disease groups (*P* < 0.05). The ultrasound scores of both lungs, left lung, right lung, bilateral lung and double basal lung in the NRDS group were significantly higher than those in the other lung disease groups (*P* < 0.05). There was a significant positive correlation between lung ultrasound score and X-ray grade (*r* = 0.841, *P* < 0.001). The area under the curve (AUC) of lung ultrasound score for the differential diagnosis of NRDS and common lung diseases was 0.907. The AUC of lung ultrasound score in the differential diagnosis of mild and moderate, and moderate and severe NRDS were 0.914 and 0.933, respectively, which had high clinical value. The lung ultrasound score was positively correlated with the level of PaCO_2_ (*r* = 0.254, *P* = 0.041), and negatively correlated with the levels of SpO_2_ and PaO_2_ (*r* = − 0.459, − 0.362, *P* = 0.001, 0.003). The AUC of successful mechanical ventilation withdrawal predicted by the pulmonary ultrasound score before extubation was 0.954 (95% CI 0.907–1.000). The predictive value of successful extubation was 10 points of the pulmonary ultrasound score, with a sensitivity of 93.33% and a specificity of 88.00%.

**Conclusion:**

The bedside lung ultrasound score can intuitively reflect the respiratory status of neonates, which provides clinicians with an important basis for disease evaluation.

## Introduction

Neonatal respiratory distress syndrome (NRDS) is a condition in which the alveoli induce progressive collapse of the alveoli due to alveolar surfactant deficiency and lung dysplasia, resulting in dyspnea in neonates. NRDS is more common in newborns 4 – 12 h after birth. According to the European NRDS guidelines in 2019, the incidence of NRDS in newborns born at 28 weeks is as high as 80%. Severe NRDS can cause apnea, which is one of the main causes of early neonatal death and seriously threatens the life and health of neonates [1, 2]. Therefore, timely and accurate diagnosis of NRDS is of great significance to the prognosis of neonates.

The previous diagnosis of NRDS mostly depends on the clinical manifestations and chest X-ray examination of the neonates, and the reliability and sensitivity of the diagnosis of neonatal NRDS are high. However, due to the radioactivity of X-ray, early exposure to radiation may adversely affect the growth and development of newborns in the rapid growth period. In addition, X-ray examination is limited by the environment and the position of the neonate, and has certain limitations for neonates who are seriously ill and cannot leave the intensive care unit. At the same time, the evaluation of the disease also needs repeated testing. Therefore, a safe and accurate inspection and evaluation method is an urgent need for the inspection of NRDS [3].

With the rapid development of imaging technology in recent years, lung ultrasound has become a hot spot in medical research. Lung ultrasound has no radiation damage, and the effect of lung exploration is relatively better for neonates with thin skin, muscle and other tissues. Lung ultrasound can be carried out at the bedside at any time, which is convenient for dynamic observation of the changes in the condition of neonates with NRDS [4]. Previous studies have demonstrated that lung ultrasound plays an important role in guiding neonatal airway management, and diagnosing respiratory diseases and adult respiratory distress syndrome [5].

In this study, the 12-zone lung ultrasound scoring method was used to scan the lungs of neonates with NRDS and analyze their sonographic characteristics through high-frequency ultrasound, so as to quantitatively evaluate the lung lesions of neonates with NRDS, and to study the correlation between lung ultrasound scores and X-ray grading. The aim of this study was to evaluate the clinical application value of the lung ultrasound scoring method, and to explore the clinical value of the lung ultrasound scoring method in evaluating the severity of NRDS and the weaning of neonates, thereby providing a more intuitive, scientific and valuable ultrasound imaging basis for guiding clinical accurate diagnosis and analyzing disease changes and follow-up treatment effects.

## Results

### Comparison of ultrasonic diagnosis of lung in neonates

**Fig. 1:**
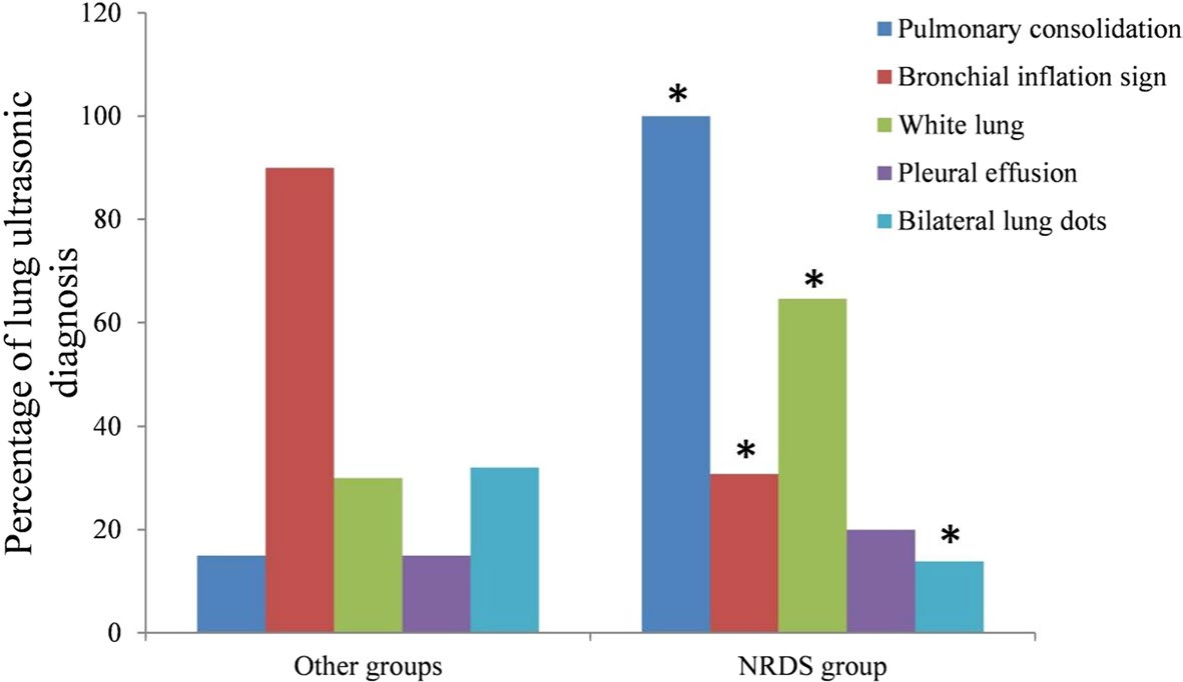
Comparison of ultrasonic diagnosis of lung in neonates. *P < 0.05 compared with the control group

The detection proportions of lung consolidation and white lung in neonates in the NRDS group were significantly higher than that in the other lung disease groups, and the detection proportions of air bronchus sign and double lung point in the NRDS group was significantly lower than that in the other lung disease groups (*P* < 0.05) (Table 1 and Fig. 1).

**Table 1:** Comparison of ultrasonic diagnosis of lung in neonates (cases, %)

Ultrasonic diagnosis	The other lung disease groups (*n* = 40)	The NRDS group (*n* = 65)	*χ*^*2*^	*P*
Lung consolidation	6 (15.00%)	65 (100.00%)	81.708	< 0.001
Aerated bronchus sign	36 (90.00%)	20 (30.77%)	34.904	< 0.001
White lung	12 (30.00%)	42 (64.62%)	11.878	0.001
Pleural effusion	6 (15.00%)	13 (20.00%)	0.418	0.518
Bilateral lung points	13 (32.50%)	9 (13.85%)	5.202	0.023

### Comparison of lung ultrasound scores in neonates

**Fig. 2:**
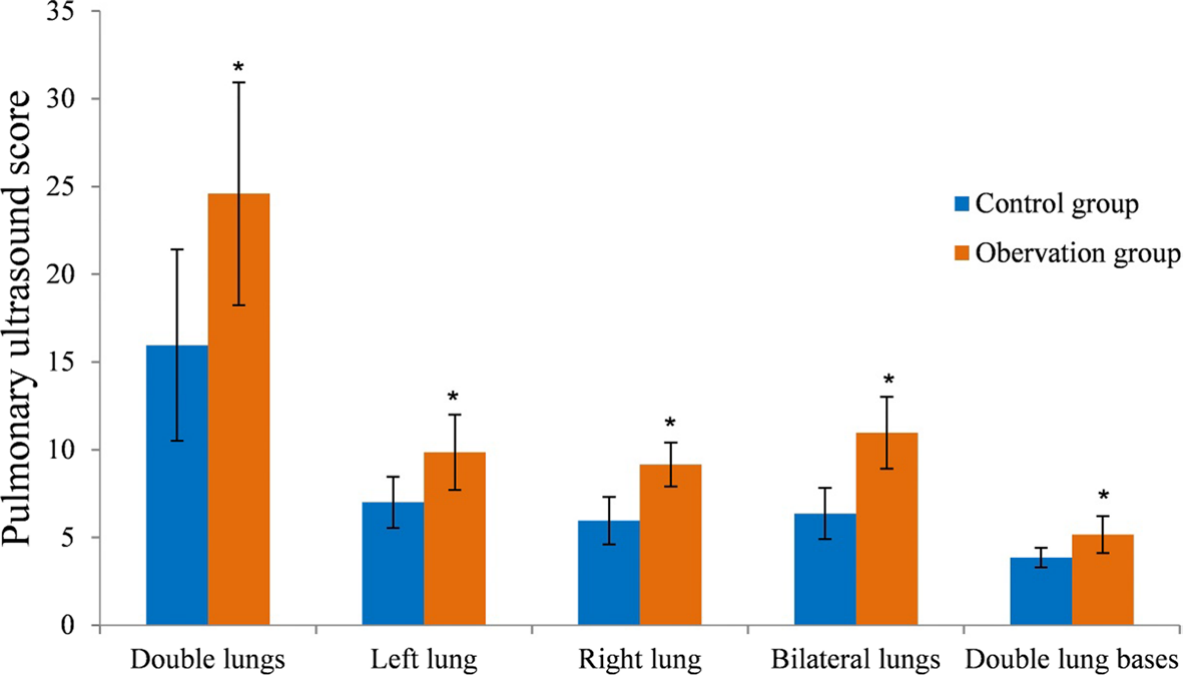
Comparison of lung ultrasound scores. *P < 0.05 compared with the control group

The patients in the NRDS group had higher pulmonary ultrasound scores of both lungs, left lung, right lung, bilateral lung, and both lung bases than the patients in the other lung disease groups (*P* < 0.05) (Table 2 and Fig. 2).

**Table 2:** Comparison of lung ultrasound scores ($\bar{X}$ ± *s*)

Groups	Both lungs	Left lung	Right lung	Bilateral lung	Both lung bases
The other lung disease groups	15.96 ± 5.46	7.01 ± 1.46	5.96 ± 1.35	6.36 ± 1.46	3.85 ± 0.56
The NRDS group	24.59 ± 6.35	9.85 ± 2.15	9.16 ± 1.25	10.96 ± 2.05	5.16 ± 1.05
*t*	7.124	7.368	12.356	12.381	7.271
*P*	< 0.001	< 0.001	< 0.001	< 0.001	< 0.001

### Correlation analysis between lung ultrasonic grading and X-ray

**Fig. 3:**
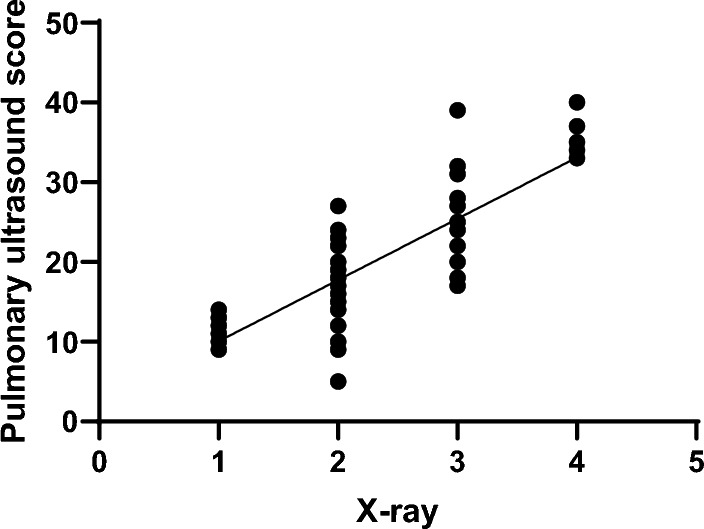
Correlation analysis between lung ultrasound score and X-ray grading

With the increase of X-ray grade, the lung ultrasound scores were gradually enhanced with significant difference between each grade group (*P* < 0.001). The results of Pearson correlation analysis showed that lung ultrasound scores were significantly positively correlated with X-ray grades (*r* = 0.841, *P* < 0.001). The distribution of lung ultrasound grading and X-ray grading is shown in Table 3. The ultrasound scores of neonates with different X-ray grades are shown in Table 4. The correlation analysis is shown in Fig. 3.

**Table 3:** Correlation analysis between lung ultrasonic grading and X-ray (cases, %)

Lung ultrasonic grading	X-ray grading				The total
	I	II	III	IV	
I	5 (7.69%)	12 (18.46%)	8 (12.31%)	0 (0.00%)	25 (38.46%)
II	1 (1.54%)	9 (13.85%)	7 (10.77%)	5 (7.69%)	22 (33.85%)
III	15 (23.08%)	3 (4.62%)	0 (0.00%)	0 (0.00%)	18 (27.69%)
The total	21 (32.31%)	24 (36.92%)	15 (23.08%)	5 (7.69%)	65 (100.00%)

**Table 4:** Correlation analysis between lung ultrasound score and X-ray grading

Grading	Cases	Lung ultrasound score (score)	F	*P*
I	21	11.10 ± 1.23		
II	24	16.08 ± 4.69	71.16	< 0.001
III	15	25.44 ± 5.21		
IV	5	35.80 ± 4.59

### Clinical value of pulmonary ultrasound score in differential diagnosis of NRDS and severity of disease

**Fig. 4:**
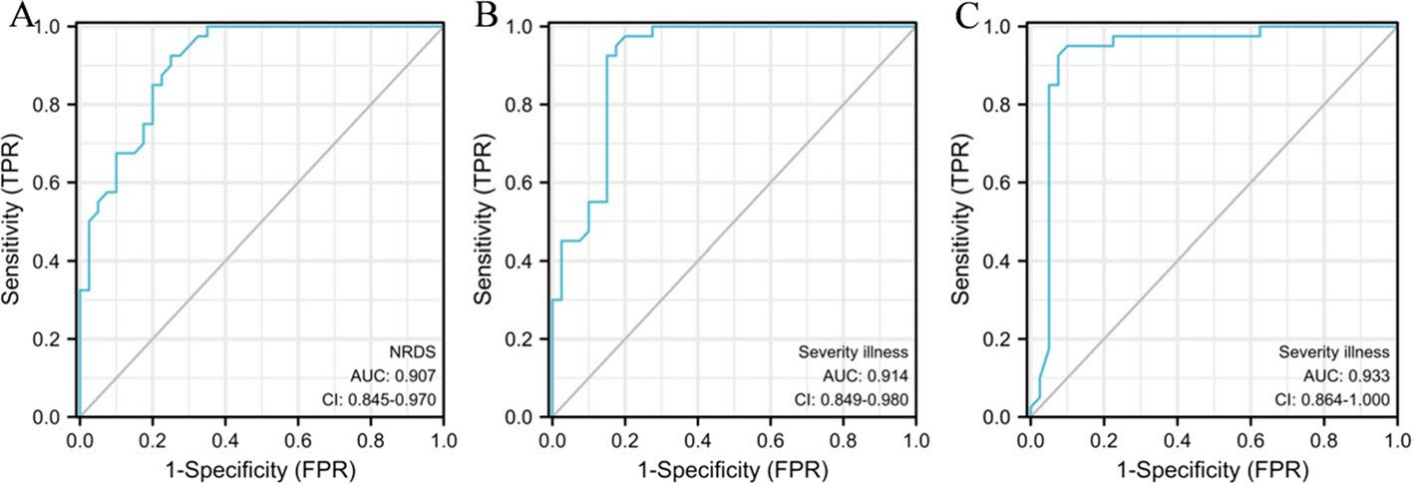
ROC curve analysis. **A** Lung ultrasound score in differential diagnosis of NRDS and common lung diseases. **B** Lung ultrasound score in differential diagnosis of mild and moderate NRDS. **C** Lung ultrasound score in differential diagnosis of moderate and severe NRDS

The AUC of lung ultrasound score in the differential diagnosis of NRDS and common lung diseases was 0.907, with a sensitivity and specificity of 89.0% and 92.5%, respectively. The AUCs of lung ultrasound score in differential diagnosis of mild versus moderate and moderate versus severe NRDS were 0.914 and 0.933, respectively, all of which had high clinical values (Table 5 and Fig. 4).

**Table 5:** Clinical value of pulmonary ultrasound score in differential diagnosis of NRDS and severity of disease

	Cut-off point	AUC	95%CI	*P*	Sensitivities	Specificities
NRDS and common lung diseases	18.5	0.907	0.845 – 0.970	< 0.001	89.0%	92.5%
Mild and moderate NRDS	27.5	0.914	0.849 – 0.980	< 0.001	91.1%	89.5%
Moderate and severe NRDS	12.5	0.933	0.864 – 1.000	< 0.001	90.0%	90.5%

### Relationship between lung ultrasound score and prognosis in neonates with NRDS

**Fig. 5:**
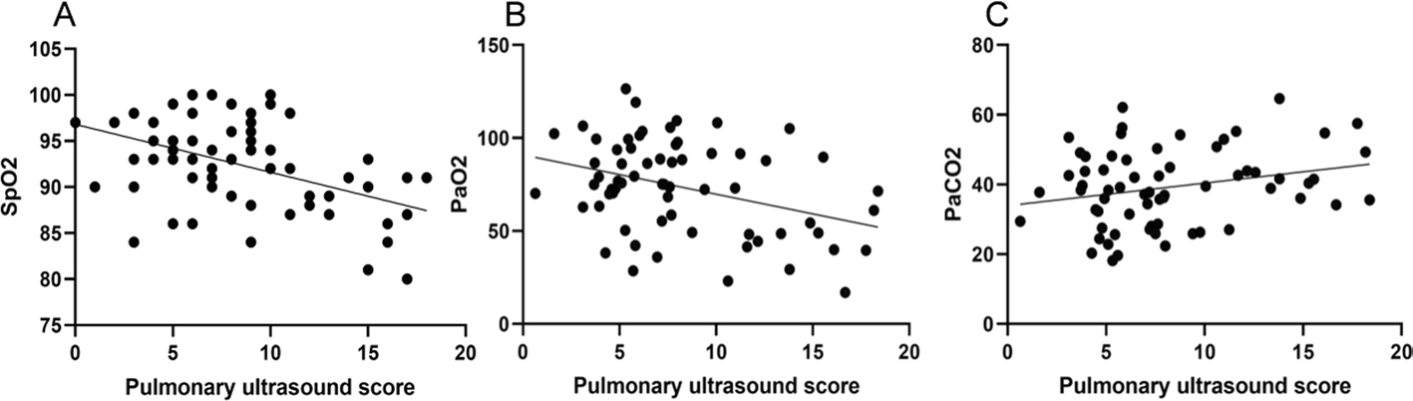
Correlation between lung ultrasound score and arterial blood gas indexes. **A** Correlation between lung ultrasound score and SpO_2_; **B** correlation between lung ultrasound score and PaO_2_; **C** correlation between lung ultrasound score and PaCO_2_

The lung ultrasound score and PaCO_2_ level of the neonates in the success group were significantly lower than these in the failure group. The level of SpO_2_ and PaO_2_ in the success group was significantly higher than that in the failure group (*P* < 0.05). The results of Pearson correlation analysis showed that lung ultrasound score was significantly positively correlated with PaCO_2_ level (*r* = 0.254, *P* = 0.041), and significantly negatively correlated with SpO_2_ and PaO_2_ level (*r* = − 0.459, − 0.362; *P* = 0.001, 0.003, Table 6 and Fig. 5).

**Table 6:** Comparison of lung ultrasound scores and arterial blood gas analysis indexes between neonates who succeeded and failed weaning ($\bar{X}$ ± *s*)

Group	Case	Lung ultrasound scores	SpO_2_ (%)	PaO_2_ (mmHg)	PaCO_2_ (mmHg)	pH value
The success group	50	6.25 ± 3.18	94.66 ± 4.32	80.42 ± 20.11	37.25 ± 12.13	7.52 ± 2.78
The failure group	15	14.66 ± 2.41	86.78 ± 5.19	50.79 ± 21.49	45.80 ± 13.21	7.48 ± 2.33
		9.441	5.912	4.928	2.346	0.051
		< 0.001	< 0.001	< 0.001	0.022	0.960

### The clinical value of ROC curve analysis of lung ultrasound score in evaluating the successful weaning from mechanical ventilation

**Fig. 6:**
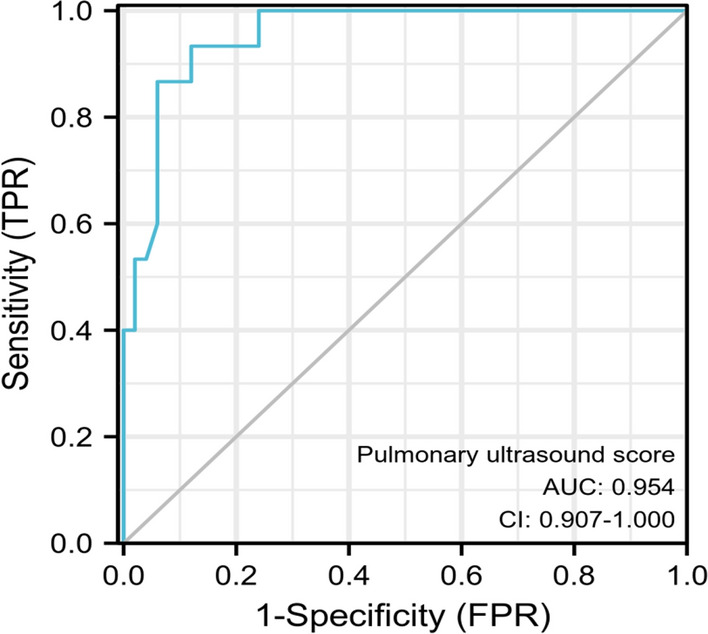
ROC curve analysis of the clinical value of lung ultrasound score in evaluating successful withdrawal from mechanical ventilation

The results of ROC curve analysis showed that the AUC of pre-extubation lung ultrasound score for predicting successful weaning from mechanical ventilation was 0.954 (95% CI 0.907–1.000), and lung ultrasound score of 10 was the predictive value of successful extubation, with a sensitivity and specificity of 93.33% and 88.00%, respectively (Fig. 6).

## Discussion

Neonates with NRDS often present with progressive dyspnea, cyanosis, respiratory failure and other symptoms in the first few hours of life. NRDS is one of the most common causes of respiratory failure and death in early neonates [6]. With the development of cesarean section in recent years, the incidence of NRDS has increased year by year, which not only seriously threatens the life safety of neonates, but also brings a heavy economic burden to families and society. Statistics from domestic scholars show that gestational age is an important factor affecting the incidence of NRDS, especially preterm infants < 35 weeks old, and the incidence of male neonates is much higher than that of female neonates. Due to the rapid progress of NRDS, early diagnosis, assessment of the severity of the disease, and effective targeted treatment are of great significance to reduce the mortality and improve their quality of life of neonates.

At present, X-ray examination is the main imaging technique for clinical diagnosis of NRDS, which can be diagnosed by observing the presence of uniform ground-glass changes in both lungs or “white lungs”. However, the specificity of X-ray examination is low, and it is difficult to diagnose the morphological distribution of lung lesions. At the same time, X-ray is radioactive, which may affect the growth and development of neonates [7, 8]. Therefore, finding a safe, reproducible and fast method for diagnosing NRDS has become the focus of medical scholars. With the rapid development of ultrasound diagnostic technology, it is possible to reflect lung lesions on ultrasound. Lung ultrasonography has the advantages of non-radioactivity, low price, bedside and repeatable, and currently has high diagnostic value in the diagnosis of atelectasis, pulmonary edema and acute lung injury [9, 10]. At the same time, due to the small lung volume and thin chest wall of newborns, the visualization operation of lung ultrasound is simpler, and lung ultrasound has certain advantages in diagnosing neonatal diseases. In this study, neonates in the NRDS group had higher proportion of patients with lung consolidation and white lung, and lower proportion of aerated bronchus sign and bilateral lung points than these in the other lung disease groups. It was also found that there was a significant correlation between lung ultrasound grading and X-ray analysis. These above results suggested that lung ultrasound had high specificity in the diagnosis of NRDS, which could effectively evaluate the changes of the condition of neonates with NRDS, and play a certain role in the early diagnosis and evaluation of the condition of NRDS. This conclusion is similar to the previous research results [11], in which it is believed that lung ultrasound has a high diagnostic value for NRDS, has higher accuracy than X-ray film, and is helpful to guide the clinical treatment of neonates. In addition, another report also reveals that lung ultrasound is a valuable diagnostic technique for monitoring and complementing chest radiographs during diagnosis and follow-up of NRDS [12].

Lung ultrasound score can evaluate the severity of lung lesions by assessing the changes in lung water content, and has become a quantitative index to judge the degree of specific lung lesions. Elsayed YN et al. [13] selects 3-day-old newborn piglets as the observation object and finds that the lung ultrasound score may have quantitative value in neonatal acute lung injury or early ARDS. At present, lung ultrasound score has been used to evaluate the severity of acute respiratory distress syndrome in adults with good validity, and many scholars believe that the lung ultrasound score can be used to evaluate the severity of NRDS in neonates [14, 15]. In the present study, neonates in the NRDS group had higher ultrasonic scores of both lungs, left lungs, right lungs, bilateral lungs and double bottom lungs than these in the other lung disease groups. The results indicated that lung ultrasound could better evaluate the lung lesions in neonates with NRDS, and had a certain help in the evaluation of lung lesions in neonates with NRDS, which could be widely used in clinical practice. However, some scholars believe that the lung ultrasound score is affected by the clinical experience of the operator, instrument probe frequency and parameters, so that the results are biased [16]. Thus, there is some controversy in the evaluation of NRDS in neonates. In addition, the results of this study also showed that the pulmonary ultrasound score gradually increased with the increase of X-ray grading, suggesting that the higher X-ray grading was, the higher ultrasound score was, and the more serious the condition of neonates was, which demonstrated that the pulmonary ultrasound score could better evaluate the pulmonary disease and severity of NRDS neonates.

Some scholars discover that [17], the ultrasound score of 12 areas of the lung can predict the severity of the disease in neonates with NRDS, and has high clinical value in the disease evaluation of neonates with NRDS. Analysis of the relevant reasons may be that the ultrasonic score of 12 areas in the lung can explore the same area of the lung from multiple directions to show different levels of lung tissue changes, and can dynamically observe the recovery of symptoms such as pulmonary edema and reduction of bronchial inflation. Therefore, bedside lung ultrasound can be used as a portable, easy-to-operate, safe, non-invasive and repeatable examination method. Some foreign scholars have found that the sensitivity of lung ultrasound in the diagnosis of neonatal atelectasis is 100%, which is significantly higher than 70% of X-ray examination, confirming the diagnostic value of lung ultrasound in neonatal lung diseases [18]. The results of this study showed that the lung ultrasound score had high clinical value in the differential diagnosis of NRDS neonates and common lung diseases, as well as the severity of NRDS. The results of this study fully confirmed the effectiveness of lung ultrasound in the diagnosis, evaluation and treatment of neonatal NRDS. In addition, the results of this study also showed that the pulmonary ultrasound score had a certain correlation with the arterial blood gas index in neonates with NRDS, and the pulmonary ultrasound score ≤ of 10 points before extubation was of high value in predicting the successful evacuation of mechanical ventilation, confirming that the lung ultrasound score was a reliable method for clinical weaning and extubation in mechanically ventilated neonates with NRDS. Wu et al. [19] showed that a lung ultrasound score of 21 or less was defined as the cut-off value for predicting the success of extracorporeal membrane oxygenation weaning. Liang et al. [20] found that the cut-off value of lung ultrasound score was 18, suggesting that pulmonary ultrasound score > 18 might be related to the failure of extubation in neonatal NRDS. Obviously, the results of this study were slightly different from these studies, considering that this was related to the small sample size of this study, the early time of the first lung ultrasound, and the interference of physiological fluids in the lungs. In addition, the subjects of this study were preterm infants, whose gestational age and weight were low, and there were factors such as congenital lung immaturity and pulmonary surfactant deficiency. All of these factors might have a certain impact on the results of the study.

To sum up, the lung ultrasound scores were gradually increased with the disease progress. The bedside lung ultrasound score could intuitively reflect the respiratory status of neonates, which provided clinicians with an important basis for disease evaluation. However, due to the limited research time and sample size, the results were analyzed on the basis of its limitations, so there might be some deviation in the results. At the same time, the ultrasound images involved in this study were not scored by two ultrasound doctors, so the image reading might be subjective. In addition, there were few kinds of diseases in neonates included in this study, and in-depth analysis of other neonatal diseases was not be carried out. Also, this experiment did not add classic lung ultrasound images. In the future, lung ultrasound images will be collected in a targeted manner, and typical ultrasound images will be provided in later research. Again, whether lung ultrasound can completely replace chest X-ray film will be further explored in the future by expanding the sample size and research time. Besides, end-tidal carbon dioxide partial pressure (PetCO_2_) can be added to the evaluation of weaning to increase the convincing results of the study.

## Materials and methods

### General materials

**Fig. 7:**
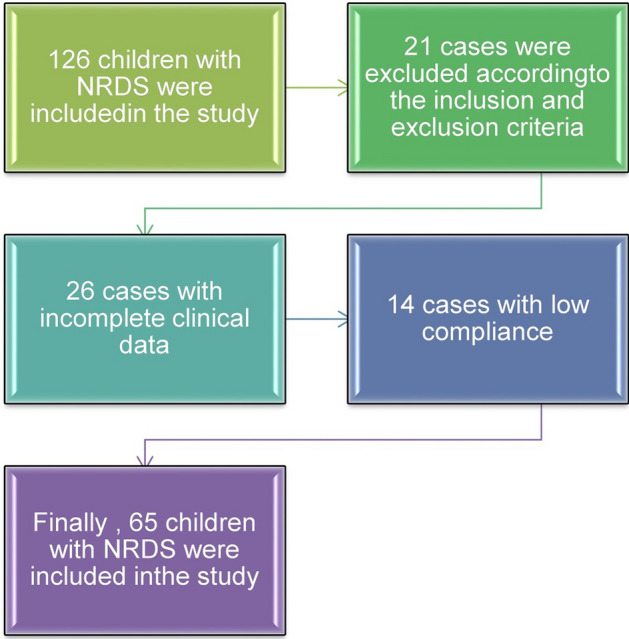
Flowchart for inclusion of 65 neonates with NRDS

The clinical data of 65 neonates with NRDS and invasive mechanical ventilation who were diagnosed in the neonatal intensive care unit of our hospital from July 2021 to July 2022 were retrospectively analyzed, and 65 neonates were included in the NRDS group. The process of general materials selection is shown in Fig. 7. Inclusion criteria: (1) All neonates met the relevant clinical diagnostic criteria for NRDS [21]; (2) The neonates were hospitalized 6 h after birth, and all were newborns; (3) The neonates and their families were informed and had good compliance. They could cooperate with the examination and treatment, and they all signed the informed consent form. Exclusion criteria: (1) The neonates with congenital dysplasia of the lung and other congenital developmental abnormalities; (2) The neonates were complicated with sepsis and other serious inflammatory diseases; (3) The neonates received the surfactant treatment; (4) The neonates with abdominal distension, pneumothorax and other restrictive pulmonary ventilation diseases; (5) The neonates with chromosomal abnormalities. During the same period, 40 neonates with other common lung diseases (neonatal wet lung and infectious pneumonia) were selected as the other lung disease groups. There were 65 cases in the NRDS group, including 36 males and 29 females, with the average gestational age of (29.85 ± 2.85) weeks, and the average birth weight of (2525.33 ± 22.46) g. The age was 3 – 28 days, with an average of (18.41 ± 4.72) days. There were 40 cases in the other lung disease groups, including 26 males and 14 females, with the average gestational age of (30.56 ± 3.62) weeks, and the average birth weight of (2613.63 ± 26.78) *g*. The age was 3 – 29 days, with an average of (17.89 ± 5.36) days. There was no significant difference in age, gestational age, gender and birth weight between the two groups (*P* > 0.05). The operation of this study was approved by the Ethics Committee of our hospital.

### Methods

Lung ultrasound examination: The ultrasound examinations were performed when the neonates were at rest. Mindray M9 bedside ultrasound diagnostic apparatus (purchased from Shenzhen Mindray Biomedical Electronics Co., Ltd.) was used for transabdominal lung ultrasound with 9–14 MHz frequency linear array probe. Instruct the neonates to take supine, lateral and prone positions, keep the neonates in a quiet state, prepare for ultrasound, keep the examination conditions relatively sterile, and adjust the ultrasound equipment to ensure its best state. With the anterior axillary front and posterior axillary line as the boundary, the lungs were divided into three areas, including anterior, lateral and posterior. Then, bounded by the line of the two nipples, each side of the viscera was divided into upper and lower lung fields, and the lungs were divided into 6 areas, including anterior and inferior, upper and lower axillary, axillary, upper and lower posterior, from top to bottom, from left to right. Save and record the ultrasound sonogram of 12 areas of both lungs in turn. All ultrasound of neonates was performed by a sonographer with more than 15 years of qualifications, and the examination results were interpreted by two sonographers. The neonate could be examined while asleep, and if the neonate was not cooperative, he or she could be given oral or enema sedation according to the doctor’s instructions. Relevant examinations could be performed when the neonate was asleep, and oral chloral hydrate or enema sedation could be given to the neonate who did not cooperate if necessary.

X-ray examination: Carestream DRXR-1 mobile X-ray machine (purchased from Shanghai Carestream Medical Equipment Co., Ltd.) was used for the X-ray examination. The hands of the neonates were instructed to be held high and fixed, and X-ray exposure was conducted when the neonates inhaled. The X-ray images of both lungs were recorded. In order to avoid errors, all X-ray examinations of neonates were performed by a radiologist with more than 15 years of qualifications, and the examination results were interpreted by two radiologists.

### Outcome measures

- (1) Lung ultrasound manifestation: The lung ultrasound manifestations of the two groups, such as lung consolidation, aerated bronchus sign, white lung, pleural effusion, and bilateral lung points, were observed.
- (2) Lung ultrasound score: Based on the transabdominal lung grading standard and the lung scoring method for NRDS by Bouhemad et al. [22], a semi-quantitative score of 12 zones in both lung of neonates with NRDS was formulated. There was a total score of 48 points with 4 points for each region. Then, the scores of each grade of ultrasound B-line and lung consolidation were assigned. The higher the score was, the more serious the lung injury in neonates was. The specific scoring methods are demonstrated in Table 7.
- (3) Lung ultrasound grading [23]: The neonate was placed in the supine position, and the probe was placed under the xiphoid process. The B-line above the diaphragm in the expiratory phase and inspiratory phase was observed by the transabdominal ultrasound. According to the B-line grading of neonates, they were grouped into grade I, II and III. The distribution of ultrasonic grading and X-ray grading of neonates in the NRDS group was analyzed. The specific classification methods are illustrated in Table 8.
- (4) The classification of X-ray diagnosis was grouped as grade I, II, III and IV [24]. The specific classification method is shown in Table 9.
- (5) Weaning and extubation results: Bedside lung ultrasonography was performed before extubation, and the neonates were divided into the success group and the failure group according to the results of weaning and extubation. Failure: Respiratory insufficiency occurred in the neonate after extubation, which was not relieved following corresponding measures, and invasive or non-invasive mechanical ventilation was resumed 48 h after weaning. The arterial blood gas analysis indexes of the neonates including percutaneous arterial oxygen saturation (SpO_2_), arterial blood oxygen partial pressure (PaO_2_), arterial blood carbon dioxide partial pressure (PaCO_2_) and arterial blood pH value were compared in the success group and the failure group. Pearson analysis was used to analyze the correlation between lung ultrasound scores and arterial blood gas indexes, and ROC curve was used to analyze the lung ultrasound to evaluate the efficacy of successful weaning from mechanical ventilation.

**Table 7:** Semi-quantitative score of 12 areas in both lungs

Lung ultrasound manifestation	Score
Smooth A-line or less than 3 isolated B-lines	0
Scattered clear B-line	1
A large number of B-lines (partial fusion)	2
A large number of fused B-lines (waterfall sign)	3
Consolidation of lung	4

**Table 8:** Lung ultrasonic grading standard

Grade	Ultrasonic manifestation
I	Obvious B-line could only be seen above the respiratory septum of the neonate
II	Obvious B-lines could only be seen above the transverse septum of the respiratory phase of the neonate, and a large number of compatible B-lines could be seen when exhaling
III	A large number of fused B-lines was seen in neonates during breathing

**Table 9:** X-ray diagnosis classification

Grade	X-ray manifestation
I	Only a wide range of small reticular particle shadows could be seen in the lung field, and the lower lobe was more obvious
II	The transparency of lung field began to decrease, with extensive reticular particle shadow, aerated bronchus sign and consolidation of both lungs
III	The shadow of fine particles in the lung field largely increased, and the transparency of the lung field decreased obviously. The aerated bronchus sign was more extensive, showing ground-glass changes
IV	The density of lung field generally increased, showing white lung and the disappearance of heart shadow

### Statistical analysis

The data were analyzed using SPSS 20.0 software. The nonparametric test (Kruskal–Wallis H) was used to test gestational age, birth weight, lung ultrasound scores and other quantitative data in accordance with normal distribution. The measurement data were expressed in (‾*x* ± *s*), and were compared using the independent samples-t test between two groups, the repeated measures analysis of variance among multiple groups, and the SNK-q test of multi-sample means between groups. The enumeration data such as the gender, lung ultrasonic performance, lung ultrasonic classification and X-ray classification were expressed in (%), and were compared using χ^2^ inspection. The correlation between ultrasonic grading and X-ray grading was analyzed using Pearson correlation analysis. The clinical value of pulmonary ultrasound score in the differential diagnosis of NRDS and the severity of the disease was analyzed using ROC curve analysis. *P* < 0.05 indicated that the statistical results were statistically significant.

## Data Availability

The datasets used and/or analyzed during the current study are available from the corresponding author on reasonable request.
